# Volumetric parameters from [ ^18^F]FDG PET/CT predicts survival in patients with high‐grade gastroenteropancreatic neuroendocrine neoplasms

**DOI:** 10.1111/jne.13170

**Published:** 2022-06-21

**Authors:** Henning Langen Stokmo, Mahmoud Aly, Inger Marie Bowitz Lothe, Austin J. Borja, Siavash Mehdizadeh Seraj, Rina Ghorpade, Xuan Miao, Geir Olav Hjortland, Eirik Malinen, Halfdan Sorbye, Thomas J. Werner, Abass Alavi, Mona‐Elisabeth Revheim

**Affiliations:** ^1^ Division of Radiology and Nuclear Medicine Oslo University Hospital Oslo Norway; ^2^ Institute of Clinical Medicine, Faculty of Medicine University of Oslo Oslo Norway; ^3^ Department of Radiology Hospital of the University of Pennsylvania Philadelphia Pennsylvania USA; ^4^ Department of Radiology Asyut University Hospital Asyut Egypt; ^5^ Department of Pathology Oslo University Hospital Oslo Norway; ^6^ Perelman School of Medicine at the University of Pennsylvania Philadelphia Pennsylvania USA; ^7^ Department of Oncology Oslo University Hospital Oslo Norway; ^8^ Department of Medical Physics Oslo University Hospital Oslo Norway; ^9^ Department of Physics University of Oslo Oslo Norway; ^10^ Department of Oncology Haukeland University Hospital Bergen Norway; ^11^ Department of Clinical Science University of Bergen Bergen Norway

**Keywords:** [^18^F]FDG PET/CT, high‐grade gastroenteropancreatic neuroendocrine neoplasia, overall survival, prognosis, volumetric parameters

## Abstract

A positive fluorine‐18 labelled 2‐deoxy‐2‐fluoroglucose ([^18^F]FDG) positron emission tomography/computed tomography (PET/CT) has been associated with more aggressive disease and less differentiated neuroendocrine neoplasms (NEN). Although a high maximum standardized uptake value (SUV_max_) predicts poor outcome in NEN, volumetric parameters from [^18^F]FDG PET have not been evaluated for prognostication in a pure high‐grade gastroenteropancreatic (GEP) NEN cohort. In this retrospective observational study, we evaluated the volumetric PET parameters total metabolic tumour volume (tMTV) and total total lesion glycolysis (tTLG) for independent prognostication of overall survival (OS). High‐grade GEP NEN patients with [^18^F]FDG PET/CT examination and biopsy within 90 days were included. Total MTV and tTLG were calculated using an adaptive thresholding software. Patients were dichotomised into low and high metabolic groups based on median tMTV and tTLG. OS was compared using Kaplan–Meier estimator and log‐rank test. Uni and multivariable Cox regression was used to estimate effect sizes and adjust for tumour differentiation and SUV_max_. Sixty‐six patients (median age 64 years) were included with 14 NET G3 and 52 NEC cases after histological re‐evaluation. Median tMTV was 208 cm^3^ and median tTLG 1899 g. Median OS in the low versus high tMTV‐group was 21.2 versus 5.7 months (HR 2.53, *p* = 0.0007) and 22.8 versus 5.7 months (HR 2.42, *p* = 0.0012) in the tTLG‐group. Adjusted for tumour differentiation and SUV_max_, tMTV and tTLG still predicted for poor OS, and both tMTV and tTLG were stronger prognostic parameters than SUV_max_. Both regression models showed a strong association between volumetric parameters and OS for both neuroendocrine tumours (NET) G3 and neuroendocrine carcinomas (NEC). OS for the tTLG low metabolic NEC was much higher than for the tTLG high metabolic NET G3 (18.3 vs. 5.7 months). High‐grade GEP NEN patients with high tMTV or tTLG had a worse OS regardless of tumour differentiation (NET G3 or NEC). Volumetric PET parameters were stronger prognostic parameters than SUV_max_.

## Graphical abstract

A high SUV_max_ predicts poor outcome in NEN, but volumetric parameters from [^18^F]FDG PET have not been evaluated for prognostication in a pure high‐grade GEP NEN cohort. In this retrospective observational study, we evaluated volumetric PET parameters for independent prognostication of OS. Sixty‐six patients were included with 14 NET G3 and 52 NEC cases after histological re‐evaluation. Adjusted for tumour differentiation and SUV_max_, tMTV and tTLG still predicted for poor OS, and both tMTV and tTLG are stronger prognostic parameters than SUV_max_.

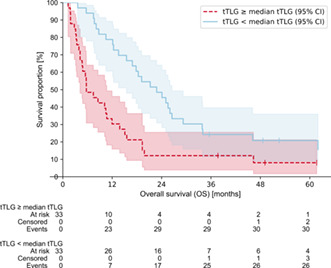

## 1 INTRODUCTION

Gastroenteropancreatic (GEP) neuroendocrine neoplasms (NEN) are a group of heterogeneous neoplasms. The 2010 World Health Organization (WHO) Classification of Tumours of the Digestive System was based on morphology and graded according to their proliferation rate: low‐grade, well‐differentiated (WD) neuroendocrine tumours (NET) (G1, G2), high‐grade, poorly differentiated (PD) neuroendocrine carcinomas (NEC) (G3). NEC could be either small cell (SC) or large cell (LC) carcinomas. The marker of proliferation antigen Ki‐67 (Ki‐67)^1^ had an index of ≤2% for NET G1, 3–20% for NET G2, and >20% for NEC.^2^ The 2019 WHO Classification of Tumours: Digestive System Tumours included an entity of WD NEN within the high‐grade category, NET G3 (Ki‐67 >20%).^3^ Hence, the term high‐grade GEP NEN now encompasses LC NEC, SC NEC and NET G3. High‐grade GEP NEN can primarily appear anywhere along the gastrointestinal (GI) tract with main predilection sites being the oesophagus, stomach, pancreas, and large intestine.^4^ Most high‐grade GEP NEN patients present with metastatic disease at the time of diagnosis and the prognosis is usually poor with limited treatment benefit and a short survival.^5^ Present knowledge of a general prognostic pattern is limited for these patients.^5^, ^6^, ^7^

Use of positron emission tomography/computed tomography (PET/CT) employing the radioactive tracer fluorine‐18 labelled 2‐deoxy‐2‐fluoroglucose ([^18^F]FDG) for staging, restaging, and monitoring treatment response has been established for many types of malignancies. [^18^F]FDG is a radiolabeled glucose analogue taken up by cells that rapidly consume and metabolise glucose, such as cancer and inflammatory cells.^8^ A significant increase in metabolic activity, as measured by uptake of [^18^F]FDG in cells, of a tumour is associated with worse prognosis in several cancers, and significant changes in metabolic activity from pre‐treatment to post‐treatment can be used in predicting response to treatment.^9^, ^10^, ^11^ Standardized uptake value (SUV) is the commonest used parameter for the quantification of metabolic activity, and maximum SUV (SUV_max_) has been most frequently used representing the maximum SUV in one voxel in a tumour.^12^, ^13^, ^14^ However, metabolic tumour volume (MTV) and total lesion glycolysis (TLG) are volumetric parameters that are obtained by using a pre‐defined threshold to delineate the tumour and its [^18^F]FDG uptake/metabolic activity.^15^ Thus, MTV and TLG are more comprehensive tools for tumour evaluation and have been shown to provide prognostic information in other cancers.^16^, ^17^, ^18^, ^19^ Further, by summing the MTV and TLG for all metabolically active tumours, we can calculate the total MTV (tMTV) and total TLG (tTLG) to gain knowledge about the patient's total tumour burden (disease burden).

Some previous studies have shown the prognostic role of different clinical and histopathological parameters on overall survival (OS) in high‐grade GEP NEN. Performance status (PS), primary tumour location, tumour morphology, Ki‐67 index, serum level of platelets and lactate dehydrogenase (LDH) have shown prognostic value for survival.^5^, ^20^, ^21^ Han et al. recently published a systematic review and meta‐analysis showing the prognostic performance of [^18^F]FDG PET/CT in NEN using visual assessment or SUV_max_ as parameters. Most studies included NEN of all grades, and the authors noted that most studies did not make a clear distinction between NET G3 and NEC. This review included 23 studies with a total of 1799 patients, and showed an adjusted hazard ratio (HR) = 3.16 for [^18^F]FDG PET/CT as prognostic for OS^22^.

[^18^F]FDG PET/CT in patients with high‐grade GEP NEN is increasingly being used, but only three studies so far have investigated the value of volumetric parameters on OS in NEN.^23^, ^24^, ^25^ One study looked at only pancreatic NET and NEC, one study looked at only gastric NEC, and one study included all tumour locations and all tumour grades. Our study aims to investigate the prognostic value of volumetric parameters (tMTV and tTLG) from [^18^F]FDG PET/CT in a pure high‐grade GEP NEN cohort.

## 2 MATERIALS AND METHODS

### 2.1 Statements of Ethics

This study was done in concordance with the Declaration of Helsinki and the Declaration of Taipei. Approval from the regional committee for medical and health research ethics (2012/490, 2012/940, 2018/1940) and the local data protection officer was obtained. Informed consent was obtained from all patients at the time of inclusion but was waived for the patients in terminal phase and deceased.

### 2.2 Patient cohort and data sources

A total of 192 patients, diagnosed and treated at Department of Oncology, Oslo University Hospital, Norway between January 2000 and July 2018, were identified retrospectively from an institutional database. These patients were participants in two multi‐institutional Nordic NEC registries organised by the Nordic Neuroendocrine Tumour Group, one previously published.^5^ Patient inclusion criteria in these NEC registries were according to the WHO 2010 tumour classification^2^ and as follows: NEC (Ki‐67 >20%), primary tumour in the GI system, or unknown primary with dominance of GI‐metastases and metastatic or noncurable locally advanced disease, primary or metastatic tumour with >30% component of NEC (G3). In addition, all patients with a [^18^F]FDG PET/CT within 90 days of their diagnosis were eligible for inclusion in the current study. Time of diagnosis was defined as the date of first biopsy. As the [^18^F]FDG PET/CT was part of the patient's clinical work‐up the recommended timing of the [^18^F]FDG PET/CT after surgery or chemotherapy could not be strictly adhered to. For all patients, data regarding basic patient characteristics, histopathology, biochemistry, diagnostic imaging, treatment, and survival were retrospectively collected from case report forms at our institution. A local patient database constructed with EpiData Manager v4.6.0.4 (EpiData Association, Odense, Denmark)^26^ contained all the study data.

### 2.3 Histological re‐evaluation

Samples were re‐collected from several different hospitals in South‐East Norway. The material consisted of surgical resection specimens from primary tumours from different origins, biopsies from the primary tumours or from metastases, and core biopsies. For some patients there were multiple samples, both from the primary tumour and metastases. All cases had been evaluated initially for histopathological tumour characteristics on haematoxylin & eosin (H&E) stained slides, and with a neuroendocrine panel of markers such as synaptophysin, chromogranin A and Ki‐67. The slides were re‐examined by an experienced pathologist according to the criteria in the 2019 WHO classification.^3^ The H&E slides were assessed according to a standardised template consisting of origin of the tumour (when possible, based on the material received), tumour growth pattern, the location of vessels close to or distant from the tumour, adjacent adenoma or adenocarcinoma, adjacent NET, desmoplastic stroma and confluent necrosis similar to what was done in the recent study by Elvebakken et al.^27^

The result of the immunohistochemical examination of synaptophysin positivity was graded as 0 (0%), 1+ (1%–33%), 2+ (34%–66%) and 3+ (67%–100%) positive tumour cells, and chromogranin A as percentage positive tumour cells. Ki‐67 labelling was also graded in percentage of positive tumour cells compared to all tumour cells. As the tumour samples available for examination differed in size the total amount of tumour cells in the slides differed, but a minimum of 600 tumour cells were counted. The tumours were then classified into PD, further divided into SC or LC NEC, and WD consistent with NET G3.

### 2.4 [ ^18^F]FDG PET/CT acquisition

All PET/CT scans were performed according to the European Association of Nuclear Medicine (EANM) guidelines^28^, ^29^ to ensure comparability between patients and measurements, and as a part of the patient's clinical work‐up. Most patients (60/66, 91%) were examined on a 40‐slice Siemens Biograph mCT hybrid PET/CT system (Siemens Healthineers), whilst the remaining patients on a Siemens Biograph 64 (4/66, 6%) and 64‐slice General Electric (GE) Discovery 690 (2/66, 3%) (GE Healthcare). The two Biograph PET/CTs were both EANM Research Ltd. (EARL)‐accredited,^30^ whilst the Discovery 690 followed similar routine quality controls harmonising with the two Biographs for cross calibration. The mean dose of [^18^F]FDG injected was 256 ± 60 MBq (6.91 ± 1.62 mCi), mean time from injection to scan was 66 ± 11 min and the mean blood glucose level was 6 ± 2 mmol/l (108 ± 36 mg/dl). Only two patients had a blood glucose level above the recommended threshold by EANM for clinical studies (11 mmol/l, 198 mg/dl).^29^ Image acquisition was performed from vertex or skull base to mid‐thighs. A low‐dose CT was performed for anatomical localisation and attenuation correction. A summary of the PET/CT scan parameters can be found in Table S1.

### 2.5 Segmentation and quantification of [ ^18^F]FDG PET/CT

**FIGURE 1:**
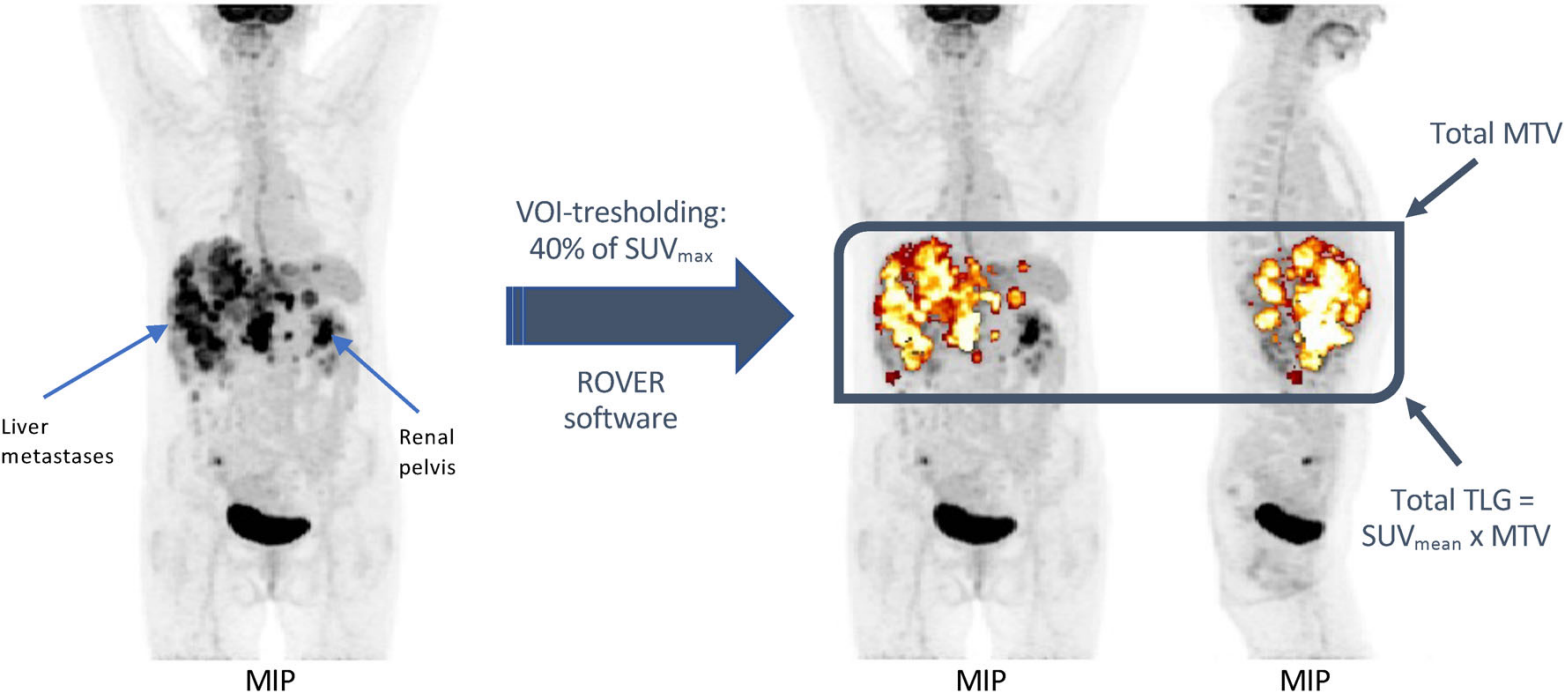
An example of a segmentation with the ROI Visualisation, Evaluation, and Image Registration (ROVER) software with an initial thresholding setting of 40% of SUV_max_. The maximum intensity projection (MIP) on the left shows a patient with multiple liver metastases from a neuroendocrine carcinoma (NEC) before segmentation. After segmentation the two MIPs on the right show that all the liver metastases have individually been segmented (coloured in gold). As we see, the renal pelvis, brain and bladder have been excluded post‐segmentation. The intense focal uptake in the right lower quadrant is not related to the patient's NEC. The metabolic tumour volume (MTV), total lesion glycolysis (TLG) together with the total MTV (tMTV) and total TLG (tTLG) can be readily calculated.

All PET/CT scans were analysed using the ROI Visualisation, Evaluation, and Image Registration (ROVER) software v3.0.5 (ABX GmbH). Defined volumes were placed over the metabolically active lesions on the PET images. The software automatically separated all lesions within these volumes into single volumes of interest (VOI) using an adaptive thresholding algorithm.^31^ The initial threshold setting for delineation for the algorithm was 40% of SUV_max._ ^32^, ^33^ For all VOIs larger than 1 cm^3^, the software automatically calculated SUV_max_, mean SUV (SUV_mean_), MTV and TLG. The MTV and TLG for all evaluated VOIs were subsequently summed to get tMTV and tTLG. All SUVs were corrected for bodyweight. All VOIs were evaluated by a radiologist and difficult cases were also evaluated by a second nuclear medicine physician. Figure 1 shows the segmentation procedure for one of our patients.

### 2.6 Statistics and data analysis

All data wrangling, plots and statistical analyses were done using Python v3.8.3 (Python Software Foundation).^34^ A list of Python packages with version number^35^, ^36^, ^37^, ^38^, ^39^, ^40^, ^41^, ^42^, ^43^, ^44^, ^45^ used can be found in Table S2. Normally distributed variables were presented as mean ± standard deviation (SD), non‐normal distributed variables as median with either interquartile range (IQR) or range, and categorical variables as numbers with percentages in parentheses. Numbers presented in the text were rounded to its nearest whole integer, whilst decimal numbers were presented in the tables. Evaluation of normality of variables was done visually using histogram plots or QQ‐plots, or statistically using the Shapiro–Wilk test. Correlation between different variables was assessed using a novel correlation coefficient Phi_K (*φ*_*K*_) allowing for correlation between mixed variable types.^46^ The coefficient only allows for positive correlations ranging from 0 to 1 and was interpreted similar to the Pearson correlation coefficient. Differences between groups were assessed using either Chi‐squared test, Fisher's exact test, Kruskal‐Wallis test, or two‐sample *t*‐test depending on the type of variable. The variables tMTV, tTLG, Ki‐67, PS, TNM‐stage were dichotomised before survival analysis. The duration of OS and median OS was assessed using the Kaplan–Meier estimator and compared using the log‐rank test. The OS was calculated as the time from diagnosis in months until date of death or date of last observation. For patients that were alive at the date of the last observation this date was used for censoring. Further, to estimate effect sizes and assess the independent effect of predictor variables on OS, univariable and multivariable analyses using Cox regression were used. The maximum number of predictor variables in the multivariable Cox model was limited to approximately ten events per variable.^47^ Cox model performance and comparison was assessed with Akaike Information Criterion (AIC), (relative) likelihood and “evidence” ratios.^48^, ^49^ Burnham et al.^48^ define the (relative) likelihood $\ell_{i}=e^{-\frac{1}{2}{∆}_{i}}$, where Δ_i_ denotes the difference in AIC between model *j* and the model with the lowest AIC. The inverse of this likelihood gives a ratio for the “best” model versus model *j*. Follow‐up time was defined using the reverse Kaplan–Meier estimator.^50^ No imputation of missing data was made, and the number of missing values for each variable can be found in Tables 1 and 2. No adjustment for multiple testing was implemented as this was considered an exploratory study. As recommended by the American Statistical Association the term “statistical significance” was not used in this manuscript.^51^, ^52^ All *p*‐values were reported as continuous quantities. Lower *p*‐values were taken as increasing evidence against the test hypothesis and the entire model (including all assumptions) used to compute it. All *p*‐values were two‐sided.

**TABLE 1:** Patient characteristics, part 1 – grouped by total MTV and total TLG

Variable name	Missing	Overall	Total MTV group		*p*‐value	Total TLG group		*p*‐value
			Low	High		Low	High	
Total, *n*		66	33	33		33	33	
Gender, *n* (%)	0				1.000			1.000
Male		36 (54.5)	18 (54.5)	18 (54.5)		18 (54.5)	18 (54.5)	
Female		30 (45.5)	15 (45.5)	15 (45.5)		15 (45.5)	15 (45.5)	
Age at diagnosis [years], median [Q1,Q3]	0	63.5 [53.8,71.0]	60.0 [53.0,70.0]	64.0 [56.0,72.0]	0.658	60.7 (12.4)	62.4 (12.9)	0.587
Age at death [years], median [Q1,Q3]	0	65.5 [57.0,73.8]	65.0 [58.0,74.0]	66.0 [57.0,73.0]	0.852	63.0 (12.2)	63.4 (12.6)	0.913
Status at end of study, *n* (%)	0				0.149			0.475
Alive		9 (13.6)	7 (21.2)	2 (6.1)		6 (18.2)	3 (9.1)	
Dead		57 (86.4)	26 (78.8)	31 (93.9)		27 (81.8)	30 (90.9)	
Body mass index [kg/m^2^], median [Q1,Q3]^a^	2	25.0 [22.8, 28.0]	25.0 [23.0, 28.0]	25.0 [22.0, 28.5]	0.681	25.0 [23.0, 28.0]	25.0 [22.0, 28.0]	0.803
Performance status (WHO), *n* (%)^a^	2				0.142			0.095
Grade 0		16 (25.0)	12 (37.5)	4 (12.5)		12 (38.7)	4 (12.1)	
Grade 1		31 (48.4)	14 (43.8)	17 (53.1)		14 (45.2)	17 (51.5)	
Grade 2		15 (23.4)	6 (18.8)	9 (28.1)		5 (16.1)	10 (30.3)	
Grade 3		1 (1.6)		1 (3.1)			1 (3.0)	
Grade 4		1 (1.6)		1 (3.1)			1 (3.0)	
Comorbidity, *n* (%)^a^	2				0.871			0.871
Yes		45 (70.3)	22 (71.0)	23 (69.7)		22 (71.0)	23 (69.7)	
No		19 (29.7)	9 (29.0)	10 (30.3)		9 (29.0)	10 (30.3)	
TNM‐staging, *n* (%)^a^	3				0.029			0.029
Clinical		49 (77.8)	20 (64.5)	29 (90.6)		20 (64.5)	29 (90.6)	
Pathological		14 (22.2)	11 (35.5)	3 (9.4)		11 (35.5)	3 (9.4)	
TNM‐stage, *n* (%)	0				0.048			0.048
Stage I–III		11 (16.7)	9 (27.3)	2 (6.1)		9 (27.3)	2 (6.1)	
Stage IV		55 (83.3)	24 (72.7)	31 (93.9)		24 (72.7)	31 (93.9)	
Primary tumour resected, *n* (%)	0				0.010			0.010
Yes		16 (24.2)	13 (39.4)	3 (9.1)		13 (39.4)	3 (9.1)	
No		50 (75.8)	20 (60.6)	30 (90.9)		20 (60.6)	30 (90.9)	
Location of metastases, *n* (%)^a^	2							
Brain		1 (1.6)		1 (3.0)	1.000		1 (3.1)	1.000
Bone		17 (26.6)	5 (16.1)	12 (36.4)	0.121	6 (18.8)	11 (34.4)	0.258
Lung		11 (17.2)	4 (12.9)	7 (21.2)	0.583	5 (15.6)	6 (18.8)	1.000
Liver		49 (76.6)	24 (77.4)	25 (75.8)	0.890	25 (78.1)	24 (75.0)	1.000
Lymph nodes		37 (57.8)	18 (58.1)	19 (57.6)	0.831	20 (62.5)	17 (53.1)	0.613
Skin		2 (3.2)		2 (6.1)	0.494	1 (3.3)	1 (3.1)	1.000
Other		9 (14.1)	5 (16.1)	4 (12.1)	0.729	5 (15.6)	4 (12.5)	1.000
Chemotherapy type, *n* (%)^a^	9				0.056			0.056
Cisplatin/etoposide		9 (15.8)	8 (29.6)	1 (3.3)		8 (29.6)	1 (3.3)	
Carboplatin/etoposide		44 (77.2)	17 (63.0)	27 (90.0)		17 (63.0)	27 (90.0)	
Temozolomide/capecitabine		2 (3.5)	1 (3.7)	1 (3.3)		1 (3.7)	1 (3.3)	
Other		2 (3.5)	1 (3.7)	1 (3.3)		1 (3.7)	1 (3.3)	
Number of courses, median [Q1,Q3]^a^	1	4.0 [2.0, 6.0]	4.0 [2.0, 6.0]	2.0 [2.0, 5.0]	0.110	4.0 [2.0, 6.0]	2.0 [2.0, 5.0]	0.134
Total MTV [cm^3^], median [Q1,Q3]	0	207.5 [65.9, 544.9]	65.8 [20.4, 99.5]	564.1 [333.4, 877.3]	<0.001	65.8 [20.4, 116.3]	564.1 [279.3, 877.3]	<0.001
Total TLG [g], median [Q1,Q3]	0	1899.4 [604.6, 5609.5]	588.0 [219.3, 945.3]	5731.8 [2634.2, 11857.2]	<0.001	588.0 [219.3, 945.3]	5731.8 [3104.5, 11857.2]	<0.001
SUV_mean_, median [Q1,Q3]	0	6.3 [4.5, 7.7]	5.8 [4.5, 7.6]	6.5 [4.9, 7.7]	0.534	5.1 [3.9, 7.2]	6.9 [5.8, 8.1]	0.005
SUV_max_, median [Q1,Q3]	0	17.9 [12.9, 25.2]	17.0 [12.2, 20.1]	20.2 [15.0, 26.8]	0.023	12.9 [10.3, 18.7]	23.6 [16.8, 33.3]	<0.001
Time from								
Diagnosis to metastasis [days], median [Q1,Q3]^a^	2	0.0 [0.0,1.0]	0.0 [0.0,15.0]	0.0 [0.0,0.0]	<0.001	0.0 [0.0,14.0]	0.0 [0.0,0.0]	0.001
Scan to diagnosis [days], median [Q1,Q3]^b^	0	22.5 [17.0,39.5]	28.0 [21.0,45.0]	19.0 [14.0,27.0]	0.001	28.0 [20.0,44.0]	20.0 [14.0,27.0]	0.021
Scan to metastasis [days], median [Q1,Q3]^ab^	2	19.5 [12.0,32.5]	22.0 [2.0,40.5]	19.0 [13.0,25.0]	0.586	19.5 [3.0,38.5]	19.5 [13.0,26.2]	0.835
Scan to primary tumour resection [days], median [Q1,Q3]^b^	0	16.5 [−59.5,36.8]	−5.0 [−106.0,45.0]	20.0 [16.5,24.0]	0.840	−5.0 [−106.0,45.0]	20.0 [16.5,24.0]	0.840
Scan to first treatment [days], median [Q1,Q3]^ab^	9	−4.0 [−9.0,‐1.0]	−7.0 [−11.5,−3.0]	‐3.0 [−6.8,‐1.0]	0.060	−7.0 [−12.0,−3.0]	‐3.0 [−5.8,‐1.0]	0.013

Abbreviations: MTV, metabolic tumour volume; SUV, standardized uptake value; TLG, total lesion glycolysis; UNL, upper normal limit; WHO, world health organization.

^a^ Because of missing values the number of patients might not be equally distributed between the groups.

^b^ Negative numbers indicates the scan was done first.

**TABLE 2:** Patient characteristics, part 2 – grouped by total MTV and total TLG

Variable name	Missing	Overall	Total MTV group		*p*‐value	Total TLG group		*p*‐value
			Low	High		Low	High	
Total, *n*		66	33	33		33	33	
Primary tumour location, *n* (%)	0				0.665			0.346
Oesophagus		8 (12.1)	6 (18.2)	2 (6.1)		6 (18.2)	2 (6.1)	
Gastric		4 (6.1)	1 (3.0)	3 (9.1)			4 (12.1)	
Gallbladder/duct		4 (6.1)	2 (6.1)	2 (6.1)		2 (6.1)	2 (6.1)	
Pancreas		10 (15.2)	5 (15.2)	5 (15.2)		5 (15.2)	5 (15.2)	
Colon		15 (22.7)	8 (24.2)	7 (21.2)		7 (21.2)	8 (24.2)	
Rectum		13 (19.7)	7 (21.2)	6 (18.2)		8 (24.2)	5 (15.2)	
Other abdominal		1 (1.5)		1 (3.0)			1 (3.0)	
CUP with dominance of GI metastases		11 (16.7)	4 (12.1)	7 (21.2)		5 (15.2)	6 (18.2)	
Ki‐67, median [Q1,Q3]	0	83.5 [56.5,92.0]	82.0 [62.0,91.0]	85.0 [56.0,92.0]	0.551	81.0 [46.0,91.0]	87.0 [66.0,92.0]	0.256
Ki‐67 before re‐evaluation, median [Q1,Q3]	2	80.0 [50.0,90.0]	80.0 [48.5,90.0]	80.0 [53.0,90.0]	0.675	77.5 [41.2,90.0]	84.5 [66.0,90.0]	0.180
Ki‐67 dichotomised, *n* (%)	0				1.000			0.240
20–54%		15 (22.7)	8 (24.2)	7 (21.2)		10 (30.3)	5 (15.2)	
≥55%		51 (77.3)	25 (75.8)	26 (78.8)		23 (69.7)	28 (84.8)	
Tumour differentiation, *n* (%)	0				1.000			0.366
WD		14 (21.2)	7 (21.2)	7 (21.2)		9 (27.3)	5 (15.2)	
PD		52 (78.8)	26 (78.8)	26 (78.8)		24 (72.7)	28 (84.8)	
Tumour morphology, *n* (%)^a^	1				0.348			0.252
NET G3		14 (21.5)	7 (21.9)	7 (21.2)		9 (27.3)	5 (15.6)	
LC		17 (26.2)	6 (18.8)	11 (33.3)		6 (18.2)	11 (34.4)	
MiNEN/LC		2 (3.1)	2 (6.2)			2 (6.1)		
SC		30 (46.2)	16 (50.0)	14 (42.4)		15 (45.5)	15 (46.9)	
MiNEN/SC		1 (1.5)	1 (3.1)			1 (3.0)		
Mixed		1 (1.5)		1 (3.0)			1 (3.1)	
Haemoglobin, *n* (%)	2				0.498			0.404
Normal		46 (71.9)	24 (77.4)	22 (66.7)		25 (78.1)	21 (65.6)	
<11 g/dl		18 (28.1)	7 (22.6)	11 (33.3)		7 (21.9)	11 (34.4)	
Platelets, *n* (%)^a^	2				0.537			0.265
Normal		49 (76.6)	25 (80.6)	24 (72.7)		27 (84.4)	22 (68.8)	
>400 × 10^9^/l		14 (21.9)	6 (19.4)	8 (24.2)		5 (15.6)	9 (28.1)	
Not done		1 (1.6)		1 (3.0)			1 (3.1)	
LDH, *n* (%)^a^	2				0.001			0.001
>2 × UNL		11 (17.2)		11 (33.3)			11 (34.4)	
>Normal ≤2 × UNL		18 (28.1)	8 (25.8)	10 (30.3)		8 (25.0)	10 (31.2)	
Normal		33 (51.6)	21 (67.7)	12 (36.4)		22 (68.8)	11 (34.4)	
Not done		2 (3.1)	2 (6.5)			2 (6.2)		

Abbreviations: CUP, cancer of unknown primary; GI, gastrointestinal; LC, large cell; MiNEN, mixed neuroendocrine non‐neuroendocrine neoplasm; MTV, metabolic tumour volume; PD, poorly differentiated; SC, small cell; TLG, total lesion glycolysis; WD, well‐differentiated.

^a^ Because of missing values the number of patients might not be equally distributed between the groups.

## 3 RESULTS

### 3.1 Study population

**FIGURE 2:**
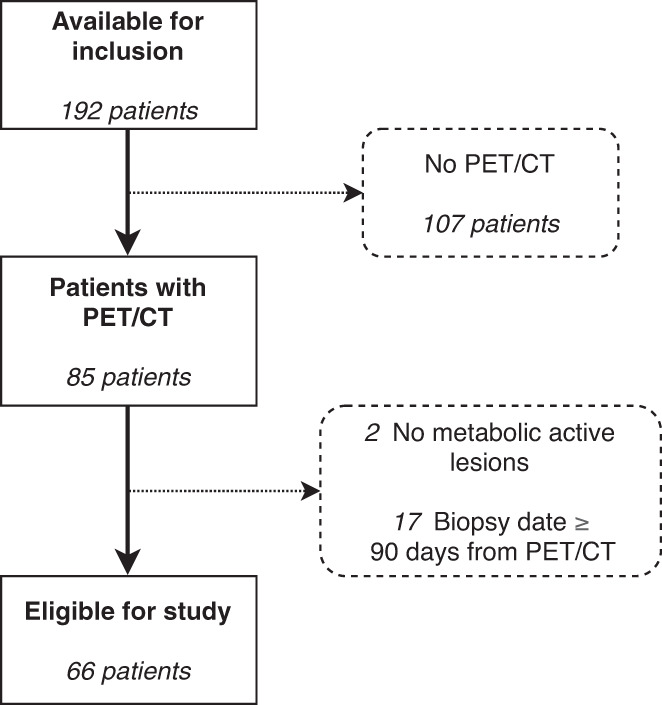
Flowchart for patient selection. Excluded patients are shown in dashed boxes.

Of the 192 patients identified, 66 patients were eligible for inclusion as shown in the patient selection flowchart in Figure 2. Of these, 36 (55%) were males and 30 (45%) females. Median age at diagnosis was 64 years. Median time between [^18^F]FDG PET/CT and diagnosis was 23 days (range 3–85 days). Most patients had stage IV at the time of diagnosis (83%) with metastases to the liver (77%) and lymph nodes (58%) predominantly, bone (27%) and lung (17%) less commonly. The most common primary tumour sites were colon (23%), rectum (20%), pancreas (15%) and oesophagus (12%). The primary tumour was resected before [^18^F]FDG PET/CT in nine (14%) patients and after in seven (11%) patients. Three of these patients had radical surgery. Most patients (73%) had a good PS of 0 or 1. There was information about use of chemotherapy treatment in 57 (86%) patients of which 53 (93%) received platinum/etoposide. Five patients (9%) received their first treatment before [^18^F]FDG PET/CT. Baseline patient characteristics are presented in Tables 1 and 2.

### 3.2 Segmentation and quantification of [ ^18^F]FDG PET/CT

Image analysis and automatic segmentation were successfully completed in all included patients. The median SUV_mean_ was 6 g/ml (range 2–20 g/ml), median SUV_max_ 18 g/ml (range 7–71 g/ml), median tMTV 208 cm^3^ (range 2–2334 cm^3^) and median tTLG 1899 g (range 22–24,845 g). The cohort was divided into a low metabolic group (tMTV < median tMTV, tTLG < median tTLG) and a high metabolic group (tMTV ≥ median tMTV, tTLG ≥ median tTLG). In the low metabolic group, median tMTV was 66 cm^3^ (range 2–196 cm^3^) and median TLG was 588 g (range 22–3104 g). In the high metabolic group, median tMTV was 564 cm^3^ (range 219–2334 cm^3^) and median TLG was 5732 g (range 1182–24,845 g).

### 3.3 Histological re‐evaluation

Re‐evaluation of histology was possible in 64 (97%) patients. One patient had re‐evaluation of Ki‐67 only, and one patient had re‐evaluation of only tumour morphology and immunohistochemistry. Re‐evaluation was based on the primary tumour in 38 (59%) cases and the metastasis in 26 (41%) cases. In general, there was no clear trend as to an over or underestimation of the Ki‐67 index but for most patients the change was approximately 5–10 percentage points after re‐evaluation. Fourteen patients (22%) were subsequently re‐classified as NET G3, whilst the remaining patients were either LC NEC (26%), SC NEC (46%) or mixed neuroendocrine non‐neuroendocrine neoplasm (MiNEN) (6%). Ki‐67 was ≥55% in 51 (77%) patients and between 20–54% in 15 (23%) patients. All re‐evaluated parameters can be found in Table S3.

### 3.4 Survival analysis

Median follow‐up time was 61.7 months (95% CI: 37.7–79.5 months) in which nine patients (13%) were alive at the end of follow‐up. For the whole cohort the median OS was 13.8 months (95% CI: 8.7–19.2 months), and estimated probability of 1‐year survival was 55% (95% CI: 41.8–65.6%) and 3‐year survival was 18% (95% CI: 10.0–28.3%).

Median OS in the tMTV low metabolic group was 21.2 months (95% CI: 15.5–26.0 months) compared to 5.7 months (95% CI: 4.2–10.7 months) in the tMTV high metabolic group (log‐rank *p* = 0.00044). Estimated probability of 1‐year survival was 79% (95% CI: 60.6–89.3%) in the tMTV low metabolic group versus 30% (95% CI: 15.9–46.1%) in the tMTV high metabolic group (log‐rank *p* = 0.00055). Estimated probability of 3‐year survival was 27% (95% CI: 11.1–39.4%) in the tMTV low metabolic group versus 9% (95% CI: 2.3–21.7%) in the tMTV high metabolic group (log‐rank *p* = 0.054).

**FIGURE 3:**
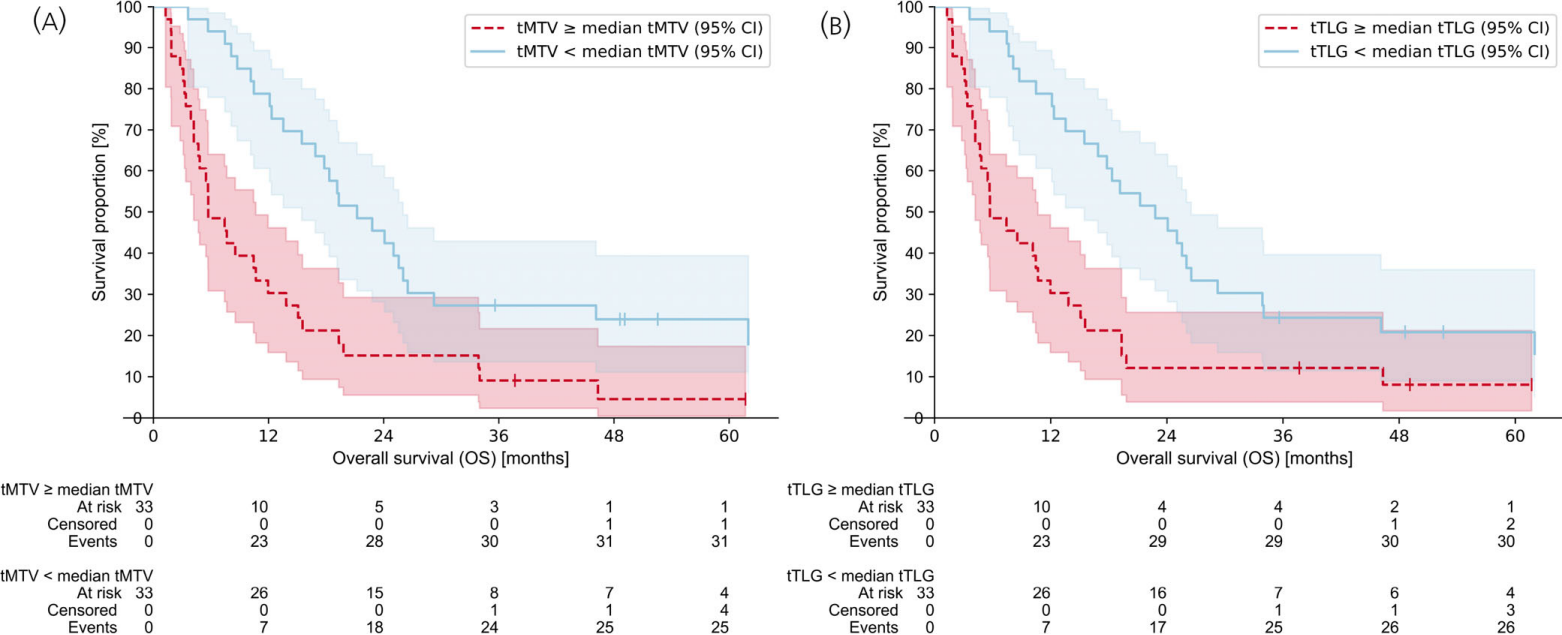
Kaplan–Meier plots with 95% compatibility intervals (CI) and “At risk”‐tables showing the overall survival (OS) for patients dichotomised into two groups. (A) Dichotomised above and below the median total metabolic tumour volume (tMTV). Patients with tMTV < median tMTV (solid light blue line) show a greater OS than patients with tMTV ≥ median tMTV (dashed red line). (B) Dichotomised above and below the total total lesion glycolysis (tTLG). Patients with tTLG < median tTLG (solid light blue line) show a greater OS than patients with tTLG ≥ median tTLG (dashed red line). Note, for both graphs the CI is large beyond 12 months because of a small number of patients at risk. Both survival curves are capped at *t* = 62 months.

Median OS in the tTLG low metabolic group was 22.8 months (95% CI: 15.5–26.5 months) compared to 5.7 months (95% CI: 4.2–10.7 months) in the tTLG high metabolic group (log‐rank *p* = 0.00091). Estimated probability of 1‐year survival was 79% (95% CI: 60.6–89.3%) in the tTLG low metabolic group versus 30% (95% CI: 15.9–46.1%) in the tTLG high metabolic group (log‐rank *p* = 0.00055). Estimated probability of 3‐year survival was 24% (95% CI: 11.4–39.6%) in the tTLG low metabolic group versus 12% (95% CI: 3.8–25.5%) in the tTLG high metabolic group (log‐rank *p* = 0.2). Kaplan–Meier plots for both tMTV and tTLG are shown in Figure 3.

Univariable analyses of OS included age, gender, PS dichotomised, TNM‐stage dichotomised, SUV_max_, tMTV, tMTV dichotomised, tTLG, tTLG dichotomised, Ki‐67, Ki‐67 dichotomised, LDH, platelets, tumour morphology, tumour differentiation, site of primary tumour and primary tumour resection. For SUV_max_ (HR 1.03, *p* = 0.003), Ki‐67 dichotomised (HR 2.56, *p* = 0.008), tumour differentiation (HR 2.68 *p* = 0.008), platelets (HR 2.44, *p* = 0.006) and PS dichotomised (HR 2.48, *p* = 0.006) there was strong evidence that these were prognostic for OS with a good clinical effect size (HR). For tMTV (HR 1.001, *p* = 0.000003), tMTV dichotomised (HR 2.53, *p* = 0.0007), tTLG (HR 1.0001, *p* = 0.0000001), tTLG dichotomised (HR 2.42, *p* = 0.001), Ki‐67 (HR 1.02, *p* = 0.0003) and tumour morphology LCNEC (HR 4.25, *p* = 0.0008) there was very strong evidence that all were prognostic for OS including a good clinical effect size. For the remaining parameters the evidence ranged from weak to very weak.

**FIGURE 4:**
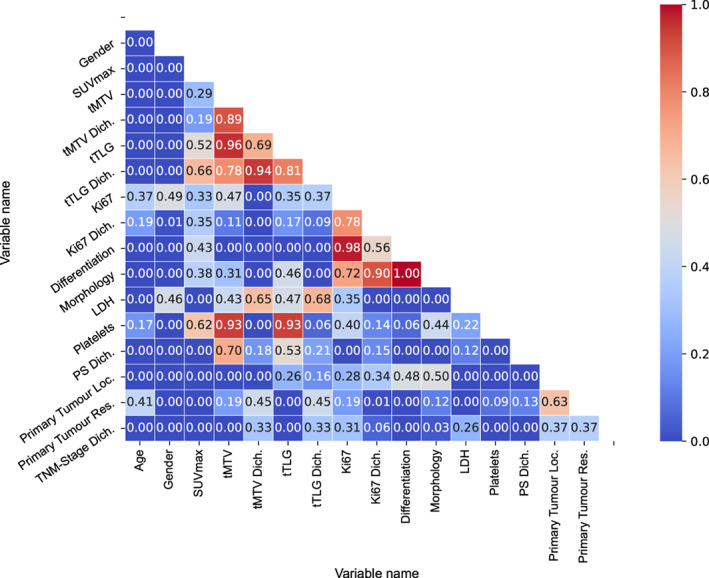
Correlation coefficient Phi_K(*φ*_*K*_) matrix visualised as a heatmap. The colour bar on the right shows the strength of the correlation from low (blue) to high (red) whilst the actual correlation coefficient is denoted within each square. The range of the correlation coefficient is from 0 to 1 and its interpretation is similar to the Pearson's correlation coefficient. Abbreviations: Dich., dichotomised; Loc., location; Res., resected.

Analysis of correlation between parameters in the univariable analyses before multivariable analysis is shown in the correlation matrix visualised as a heatmap in Figure 4. Because of their high correlation, tMTV and tTLG were analysed in separate multivariable analyses. In the multivariable analyses we included tumour differentiation and SUV_max_ as possible confounders for tMTV and tTLG as these are known strong prognostic parameters for OS. Other known prognostic parameters (platelets, LDH, PS, Ki‐67, tumour morphology) were either highly correlated to tMTV or tTLG, or tumour differentiation and were left out of the multivariable analyses. Similar HRs, *p*‐values, and compatibility intervals (CI) are expected for highly correlated variables. In both multivariable analyses tMTV and tTLG both still showed strong evidence as independent predictors correcting for SUV_max_ and tumour differentiation.

In addition; we also constructed three separate multivariable models with SUV_max_ and tumour differentiation, tMTV and tumour differentiation, and tTLG and tumour differentiation to compare the performance of these three variables. AIC and likelihood for the SUV_max_/differentiation‐model was 390.1 (model #1), for the tMTV/differentiation‐model 374.6 (model #2), and for the tTLG/differentiation‐model 368.7 (model #3). Computing the likelihood ratio, we get ℓ_model 2vs1_ = 0.000431 and ℓ_model 3vs1_ = 0.0000225. Thus, the evidence for model #2 is 2320 times stronger than for model #1, and the evidence for model #3 is 44,444 times stronger than for model #1 given our data. The result from the regression analyses is presented in Table 3.

**TABLE 3:** Results from the uni and multivariable analyses – grouped by total MTV and total TLG

Covariate	Univariable		Multivariable			
			Total MTV model		Total TLG model	
	HR (95% CI)	*p*‐value	HR (95% CI)	*p*‐value	HR (95% CI)	*p*‐value
Age	1.03 (1.002–1.05)	0.036				
Gender						
Male vs. female	1.29 (0.76–2.18)	0.351				
Performance status (WHO) dichotomised						
Grade ≥1 vs. grade <1	2.48 (1.31–4.71)	0.006				
TNM‐stage dichotomised						
Stage IV vs. stage I–III	1.66 (0.80–3.44)	0.17				
SUV_max_	1.03 (1.01–1.05)	0.003	1.03 (1.003–1.05)	0.02	1.017 (0.99–1.04)	0.13
Total MTV	1.001 (1.0006–1.002)	3.4E‐06	1.001 (1.0007–1.0016)	3.1E‐07		
Total MTV dichotomised						
High vs. low group	2.53 (1.48–4.32)	0.0007				
Total TLG	1.0001 (1.00007–1.0002)	1.4E‐07			1.00013 (1.00008–1.00017)	2.93E‐08
Total TLG dichotomised						
High vs. low group	2.42 (1.42–4.13)	0.0012				
Ki‐67	1.02 (1.01–1.04)	0.0003				
Ki‐67 dichotomised						
≥55% vs. 20–54%	2.56 (1.28–5.13)	0.008				
LDH						
>2 × UNL vs. normal	2.06 (0.99–4.28)	0.053				
>Normal ≤2 × UNL vs. normal	1.20 (0.65–2.21)	0.566				
Platelets						
>400 × 10^9^/l vs. normal	2.44 (1.29–4.62)	0.006				
Tumour morphology						
LC vs. NET G3	4.25 (1.82–9.89)	0.0008				
SC vs. NET G3	2.18 (1.01–4.68)	0.046				
Tumour differentiation						
PD vs. WD	2.68 (1.30–5.54)	0.008	2.69 (1.27–5.72)	0.01	3.01 (1.40–6.47)	0.0047
Site of primary tumour						
Oesophagus vs. CUP	1.22 (0.45–3.31)	0.69				
Gastric vs. CUP	2.73 (0.82–9.10)	0.1				
Gallbladder/duct vs. CUP	1.01 (0.27–3.76)	0.98				
Pancreas vs. CUP	0.93 (0.36–2.43)	0.89				
Colon vs. CUP	1.54 (0.66–3.62)	0.32				
Rectum vs. CUP	1.22 (0.51–2.92)	0.65				
Other abdominal vs. CUP	8.64 (0.97–74.98)	0.05				
Primary tumour resected						
No vs. yes	1.26 (0.69–2.31)	0.45				

Abbreviations: MTV, metabolic tumour volume; TLG, total lesion glycolysis; HR, hazard ratio; CI, compatibility interval; SUV, standardized uptake value; WHO, world health organization; LDH, lactate dehydrogenase; UNL, upper normal limit; NET, neuroendocrine tumour; SC, small cell; LC, large cell; PD, poorly differentiated; WD, well‐ differentiated; CUP, cancer of unknown primary.

**FIGURE 5:**
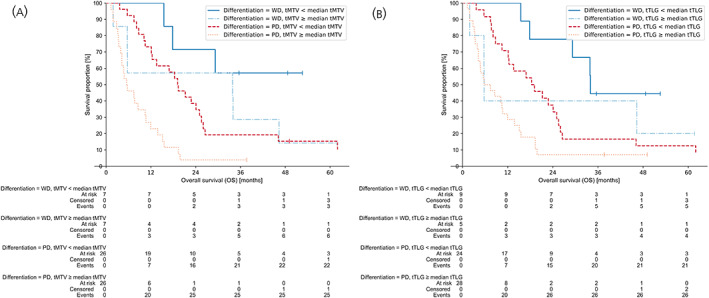
Kaplan–Meier plots showing the overall survival (OS) with “At risk”‐tables for patients dichotomised into four groups. (A) The solid blue line shows the OS when total metabolic tumour volume (tMTV) is less than the median tMTV and the tumour differentiation is well‐differentiated (WD). Similarly, the dashdotted light blue line shows the OS when tMTV is equal to or above the median tMTV and the tumour differentiation is WD. The dashed red line and dotted gold line show the OS for below and above the median tMTV given the tumour differentiation is poorly differentiated (PD). (B) Shows exactly the same as in (A), but for the variable total total lesion glycolysis (tTLG). Survival curves are capped at *t* = 62 months.

To investigate the difference in OS between patients with NET G3 and NEC, Kaplan–Meier curves for each of the variables tMTV and tTLG with low and high metabolic tumour groups were constructed (Figure 5). Median OS in the tMTV low metabolic group for NET G3 was not reached (95% CI: 15.5 – infinite months) compared to 33.9 months (95% CI: 1.9–46.3 months) in the high metabolic group for NET G3. Median OS in the tMTV low metabolic group for NEC was 19.2 months (95% CI: 12.1–25.0 months) compared to 5.5 months (95% CI: 3.9–10.5 months) in the high metabolic group for NEC. Median OS in the tTLG low metabolic group for NET G3 was 34.0 months (95% CI: 15.5 – infinite months) compared to 5.7 months (95% CI: 1.9 – infinite months) in the high metabolic group for NET G3. Median OS in the tTLG low metabolic group for NEC was 18.3 months (95% CI: 10.5–25.0 months) compared to 5.7 months (95% CI: 4.2–10.7 months) in the high metabolic group for NEC. Corresponding simulated survival curves from the multivariable Cox regression analyses are shown in Figure S1 illustrating the effects of different tumour differentiation, and different values of tMTV and tTLG on OS for the models.

## 4 DISCUSSION

In the current study we have shown that the volume‐based, semi‐quantitative PET parameters tMTV and tTLG have prognostic value in relation to OS in patients with high‐grade GEP NEN. The parameters were prognostic for both NET G3 and NEC. Further, we have shown that tMTV and tTLG are stronger parameters than SUV_max_ in predicting OS.

Traditionally, SUV_max_ has been the quantitative parameter used in studies involving PET. In the past 15 years, volume‐based PET quantification metrics such as MTV and TLG have been introduced. MTV and TLG are metabolic based volumetric parameters reflecting the tumour burden and metabolic activity, and it is reasonable to assume that a high tumour burden and a high metabolic activity indicate a more aggressive disease. Both MTV and TLG have already shown promise in several other types of malignancies,^53^ but studies with tMTV and tTLG are scarce compared to studies with MTV and TLG. Further, some studies use tMTV and MTV interchangeably or other notations making it harder to identify studies with tMTV and not lesion‐based MTV.^23^, ^24^ In a recent editorial Høilund‐Carlsen et al. advocated that a global disease score (tMTV and tTLG) must replace SUV_max_ as a quantitative parameter. Simple parameters like SUV_mean_, SUV_max_ or SUV_peak_ do not reflect the total tumour burden of the disease like tMTV and tTLG^54^. As TLG is just MTV multiplied by SUV_mean_, for many malignancies they would most likely be highly correlated and have similar prognostic value. With more advanced imaging analysis software packages becoming available, the possibility of quick and easy VOI‐delineation makes calculation of more advanced quantitative parameters like tMTV and tTLG easier. In a busy clinical practice, it is paramount that the calculation of such quantitative parameters is quick and easy to perform.

To our knowledge, this is the first study to evaluate the prognostic value of tMTV and tTLG in a high‐grade GEP NEN patient cohort according to the new 2019 WHO classification system. As far as we know, only one other study has looked at the prognostic value of tMTV and tTLG in a population of patients with NEN which included all tumour sites and all tumour grades.^23^ With a median tMTV of 4.83 cm^3^ and median tTLG of 29.5 g, they showed that patients with tMTV or tTLG higher than median tMTV or tTLG had poorer OS. Similar to our study, they found a mean tMTV of 337 cm^3^ in their subgroup of 25 patients with advanced GEP NEC, but no further analysis of this group was performed. Further, in line with our study, they also found that tMTV and tTLG were better predictors of OS compared to SUV_max_. Interestingly, even though they included different tumour sites and grades, the HRs for tMTV (HR 1.0013) and tTLG (HR 1.0002) were comparable to our study. Contrary to our study they included patients with a tMTV of 0 (G1 and G2 tumours may have no [^18^F]FDG uptake), but in a separate analysis they excluded patients with tMTV of 0 and got a higher median tMTV of 33 cm^3^.

Lim et al. reported the prognostic value of [^18^F]FDG PET/CT in 27 patients with gastric NEC and mixed adenoneuroendocrine NEC (MANEC). Like our study, they found a better OS in patients with a low tMTV/tTLG compared to a high tMTV/tTLG using receiver operating characteristic (ROC)‐derived 107.9 cm^3^/612 g for dichotomisation. Their median tMTV was 56.6 cm^3^ and median tTLG was 344.8 g which is lower than ours. However, they only had 12 (44%) patients with stage IV at diagnosis which could explain the lower median tMTV and tTLG.^24^ Furthermore, Kim et al. looked at 20 patients with pancreatic NEN and found primary tumour MTV to predict poorer OS. Only four (20%) patients had WHO 2010 G3 tumours and four patients where stage IV (not the same patients).^25^ Most studies applying [^18^F]FDG PET/CT in NEN include all tumour grades without doing subgroup analysis based on tumour grade. This is to be expected as high‐grade GEP NEN are uncommon, but it makes it difficult to extrapolate the results to high‐grade GEP NEN. As such, more studies on [^18^F]FDG PET/CT and high‐grade GEP NEN are needed to assess the value of volumetric parameters in [^18^F]FDG PET/CT in this patient group which has the poorest OS of NEN.

According to the latest European Neuroendocrine Tumour Society (ENETS) imaging guideline somatostatin receptor imaging (SSRI) is currently recommended, and commonly used, for staging and restaging NET. It is not currently recommended to routinely use [^18^F]FDG PET/CT in the evaluation of NEC, but it might be considered in G2 tumours with a high Ki‐67 or NEC.^55^

For tumours with a Ki‐67 >10% some authors recommend [^18^F]FDG PET/CT in all patients.^56^, ^57^, ^58^ In the latest Nordic GEP NEN 2021 guideline, [^18^F]FDG PET/CT is now recommended in localised NET G3 and NEC, and may be considered in G1 and G2 tumours for prognostication and therapy planning.^59^ The National Comprehensive Cancer Network (NCCN) 2021 Clinical Practice Guidelines in Oncology for Neuroendocrine and Adrenal Gland Tumours^60^ state that [^18^F]FDG PET/CT can be performed in patients with NET G3 tumours if SSRI is negative. For NEC, the guideline state that [^18^F]FDG PET/CT can be performed where clinically indicated, and routinely in NEC in the neck and thorax region. Although there are differences in the current guidelines regarding the use of [^18^F]FDG PET/CT in high grade GEP NEN, it seems to be an increasing trend from previous guidelines as to the use of [^18^F]FDG PET/CT in these tumours.

**FIGURE 6:**
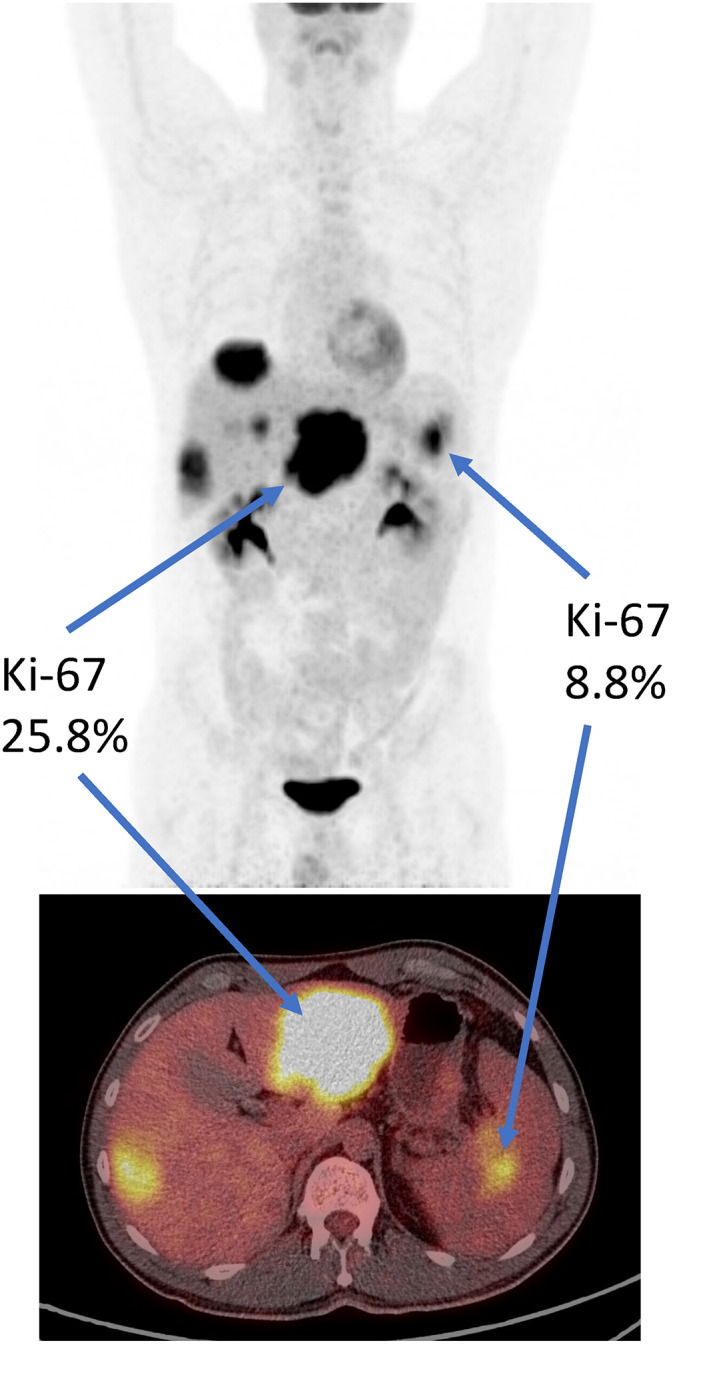
A 40‐year‐old male presented with multiple liver lesions and a possible primary tumour in the tail of the pancreas presented as a maximum intensity projection (MIP) with a fusioned axial PET/CT. He was diagnosed with a neuroendocrine carcinoma (NEC) based on a biopsy from one of the liver lesions (Ki‐67 = 25.8%). On chemotherapy, the liver metastases showed complete metabolic response, and he later had a resection of the primary tumour in the pancreas (Ki‐67 = 8.8%). On histology re‐evaluation the liver metastasis was reclassified as a well‐differentiated (WD) neuroendocrine tumour (NET) G3 (Ki‐67 = 20%).

Most high‐grade GEP NEN have metastatic disease at the time of diagnosis and patients with sufficient PS will receive chemotherapy regardless, questioning the added clinical value of [^18^F]FDG PET/CT. However, we have shown the prognostic value of tMTV and tTLG on OS in multivariable analysis, and further studies should investigate if [^18^F]FDG PET/CT also can predict treatment response in these patients. Moreover, a single biopsy from a single tumour does not reflect the known heterogeneity of this disease. The Ki‐67 in the primary tumour can be quite different from that of the metastases, and it can vary between metastases and also along the disease course.^61^, ^62^, ^63^ In our cohort many of the patients received surgery either before or after [^18^F]FDG PET/CT despite having stage IV disease at the time of diagnosis. Only 20/64 (31%) patients had their diagnosis established from a liver metastasis. Some patients would only be diagnosed with NEC post‐surgery either because of its emergent nature (e.g., ileus) or incidentally after removing a polyp during colonoscopy. Further, some patients despite being in a palliative setting had the option for surgery because of the generally aggressive surgical approach we have in Norway to prolong survival in these patients. Figure 6 shows an example from our cohort of a patient diagnosed with a primary pancreatic NET with Ki‐67 = 8.8% (resected specimen), and with Ki‐67 = 25.8% from one of several liver metastases (core biopsy). This patient was diagnosed based on the core biopsy of the liver metastasis, and consequently received chemotherapy with a good initial response. Based on this, the patient later received radical surgery of the pancreatic tumour with a curative intent. Metabolic information derived from [^18^F]FDG PET/CT regarding heterogeneity of the disease could give additional information, and suggest correct treatment and prognosis based on the correct tumour grade. It could also guide biopsies to the most metabolically active tumour or part of the tumour for more accurate tissue sampling.

Our data included patients with both NET G3 and NEC, and our analyses show that the results are valid for both tumour entities. Interestingly, looking at the patients with NET G3 separate from the patients with NEC (Figure 5) they have a different median OS based on tMTV and tTLG. Patients with NET G3 and a low tMTV or tTLG clearly show the highest median OS. Patients with NET G3 and a high tMTV or tTLG seem to have a poorer OS, like patients with NEC irrespective of their tMTV or tTLG. This is even clearer from the tTLG parameter (Figure 5B) than the tMTV parameter (Figure 5A). The low number of patients with NET G3 per group though prevents us from drawing any conclusions and further studies could give more conclusive evidence. For patients with locally advanced/metastatic NET G3 the NCCN 2021 guideline^60^ suggests to divide NET G3 into two groups – a favourable biology group with relatively low Ki‐67 and positive SSR‐based PET, and an unfavourable biology group with relatively high Ki‐67 and negative SSR‐based PET. Our data show that tMTV or tTLG could also be considered an additional parameter to group patients with NET G3 into different prognostic groups. In patients with GEP NEC prognosis is usually grim with a median survival 11–12 months after 1‐line chemotherapy.^5^ Our study shows a subgroup within GEP NEC with a much better prognosis. In our patients with NEC with a low tMTV or tTLG, median OS was as high as 18–19 months.

This study has both strengths and limitations. It was a retrospective observational study which included 66 patients, but the majority of the [^18^F]FDG PET/CT investigations were done on the same scanner and patients were from a single institution. This minimises variation in diagnostic imaging, pathology, and treatment. We restricted the inclusion of patients with less than 90 days between the [^18^F]FDG PET/CT and date of diagnosis, but optimally the time interval should be as short as possible. Although, the median with the IQR shows that many patients had the scan close to their diagnosis. In fact, only 10 patients had their [^18^F]FDG PET/CT more than 45 days after their diagnosis. Only one patient had known brain metastases at the time of diagnosis. We know that [^18^F]FDG PET/CT is not optimal for this evaluation because of the inherent high glucose metabolism in the brain. Hence, some patients could have a lower tMTV/tTLG than estimated because these are missed during segmentation, but for most patients it would probably not affect the volumetric parameters significantly. Sixteen patients had surgery before or after [^18^F]FDG PET/CT and most of them were included in the low metabolic group, five patients had started chemotherapy treatment before [^18^F]FDG PET/CT and some stage mixture existed at the time of diagnosis. These factors could clearly influence OS. However, the univariable analysis of primary tumour resection and TNM‐stage showed low evidence for this. The five patients receiving chemotherapy before the [^18^F]FDG PET/CT all had metastatic disease at the time of their diagnosis, and all got the same chemotherapy treatment. Three belonged to the low metabolic group, however four out of five still had an OS less than 20 months. One could argue that some of these patients could have ended up in the high metabolic group if the [^18^F]FDG PET/CT was done prior to treatment.

Since the follow‐up period spanned over several years, the patients got different lines of treatment during the observation period which also could influence OS. Despite this, most patients were treated with similar chemotherapy. The histopathology was systematically re‐evaluated by an experienced pathologist for consistency. Moreover, the number of cases with NET G3 were relatively few. Lastly, the biopsy site and Ki‐67 index were from both primary tumours and metastases which we know could have very different values.

Several previous studies have shown the prognostication of several different parameters on OS in NEC. Sorbye et al. showed in a multivariable model that PS, primary tumour location, serum levels of platelets, Ki‐67 and LDH were strong prognostic factors for survival.^5^ Walter et al. similarly showed in a multivariable subgroup analysis of metastatic GEP NEC, the prognostic performance of PS and number of metastatic sites.^6^ Moreover, Milione et al.^20^ showed prognostic performance for tumour differentiation, tumour stage and Ki‐67. Lastly, Heetfeld et al. and others have shown longer survival in patients with NET G3 than those with NEC.^21^, ^27^ As we have shown in the current study, after adjusting for tumour differentiation and SUV_max_, tMTV and tTLG still remain independent predictors of OS and the result is in line with previous similar studies. Further studies on independent cohorts should be performed to validate the results from our study.

## 5 CONCLUSION

A high value of total MTV or total TLG is a poor independent prognostic parameter of OS in patients with high‐grade GEP NEN adjusting for other known prognostic parameters. Both total MTV and total TLG are stronger parameters than SUV_max_ in predicting OS. Total MTV and total TLG were prognostic for both NET G3 and NEC and could be useful to categorise a poor prognostic group in the newly established NET G3 entity.

## AUTHOR CONTRIBUTIONS

**Mahmoud Aly:** Investigation; resources; writing – original draft; writing – review and editing. **Inger Marie Bowitz Lothe:** Data curation; formal analysis; investigation; methodology; resources; writing – original draft; writing – review and editing. **Austin Borja:** Investigation; resources; writing – review and editing. **Siavash Mehdizadeh Serajc:** Investigation; resources; writing – review and editing. **Rina Ghorpade:** Investigation; resources; writing – review and editing. **Xuan Miao:** Investigation; resources; writing – review and editing. **Geir Hjortland:** Data curation; funding acquisition; investigation; resources; writing – review and editing. **Eirik Malinen:** Formal analysis; methodology; writing – review and editing. **Halfdan Sorbye:** Data curation; funding acquisition; investigation; methodology; resources; writing – original draft; writing – review and editing. **Thomas J. Werner:** Investigation; project administration; resources; writing – review and editing. **Abass Alavi:** Conceptualization; project administration; writing – review and editing. **Mona‐Elisabeth Revheim:** Conceptualization; funding acquisition; investigation; project administration; resources; writing – original draft; writing – review and editing.

## CONFLICT OF INTEREST

H.L.S have received consultancy fees from Siemens Healthineers.

### PEER REVIEW

The peer review history for this article is available at https://publons.com/publon/10.1111/jne.13170.

## Supporting information


**Figure S1** Simulated overall survival (OS) plots from the multivariable Cox regression for the two variables, total metabolic tumour volume (tMTV) and total total lesion glycolysis (tTLG). The simulated survival curves show the effect of varying the variables “tMTV” and “tumour differentiation” whilst setting the variable “SUV_max_” to its median value. This to illustrate the impact of a covariate given the Cox regression model. Note that these simulated OS curves include the same number of patients (*n* = 66) and events (*n* = 57), whilst in reality the patients and events are distributed between the different curves as one would see in a Kaplan–Meier plot. A, The solid blue line shows the predicted OS when tMTV is equal to the median value (66 cm^3^) in the low metabolic group and the tumour differentiation is well‐differentiated (WD). Similarly, the dashdotted light blue line shows the OS when tMTV is equal to the median value (564 cm^3^) in the high metabolic group and the tumour differentiation is WD. The dashed red line and dotted gold line show the OS in both the low and high metabolic groups, respectively given the tumour differentiation is poorly differentiated (PD). B, Shows exactly the same thing as in (A), but for the variable tTLG. C, D; Shows the effect of varying the value of tMTV and tTLG over a whole range of values given that the tumour differentiation is PD. E, F; Similarly, these graphs show exactly the same as in (C) and (D), but given that the tumour differentiation is WD.

**Table S1** Scanner parameters – grouped by total MTV and total TLG

**Table S2** Python packages used for data wrangling and data analyses

**Table S3** Re‐evaluated histology – grouped by total MTV and total TLG

## Data Availability

The data are not publicly available due to restrictions from the hospital's Data Protection Officer.
