# Supplementary figures and images for: Volumetric parameters from [ 18F]FDG PET/CT predicts survival in patients with high‐grade gastroenteropancreatic neuroendocrine neoplasms
Source: J Neuroendocrinol. 2022 Jun 21;34(7):e13170. doi: 10.1111/jne.13170 (PMC9539477; doi:10.1111/jne.13170)

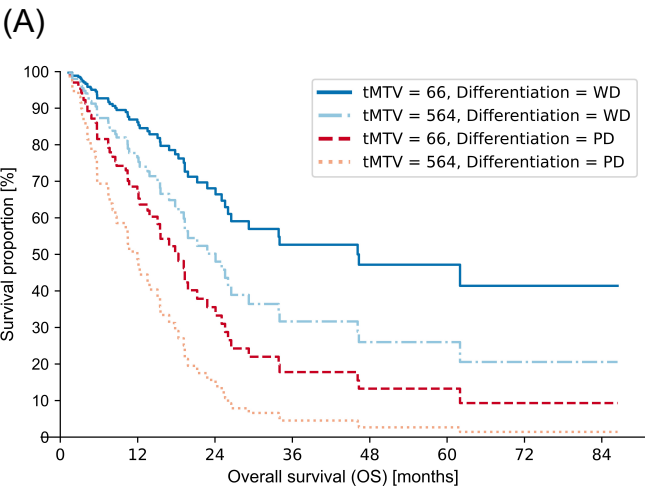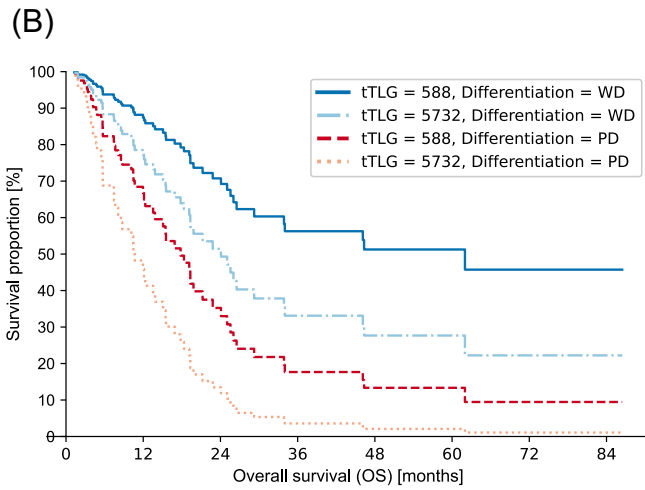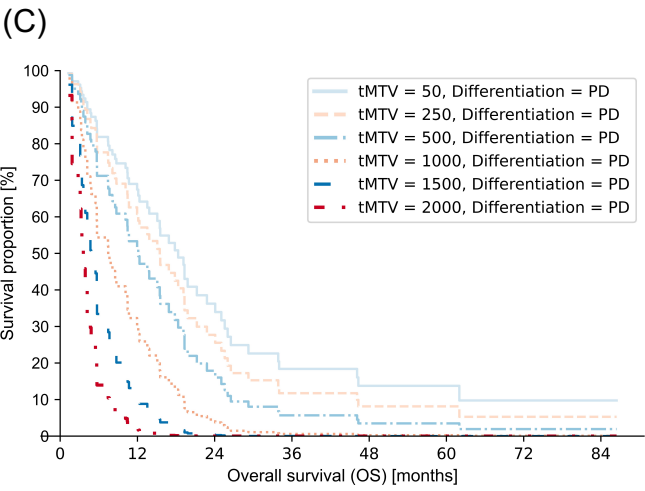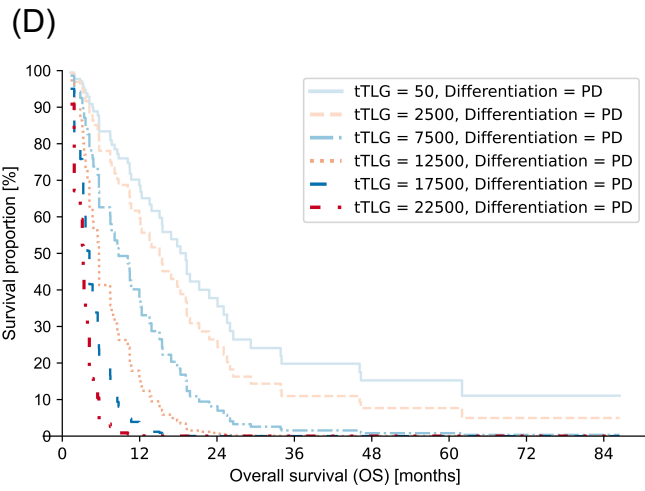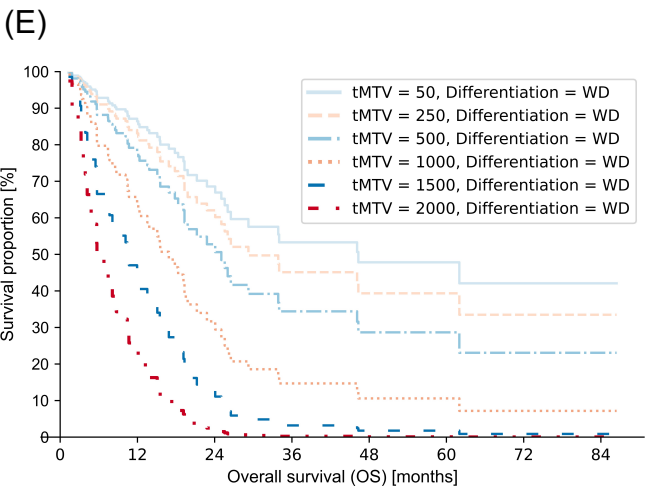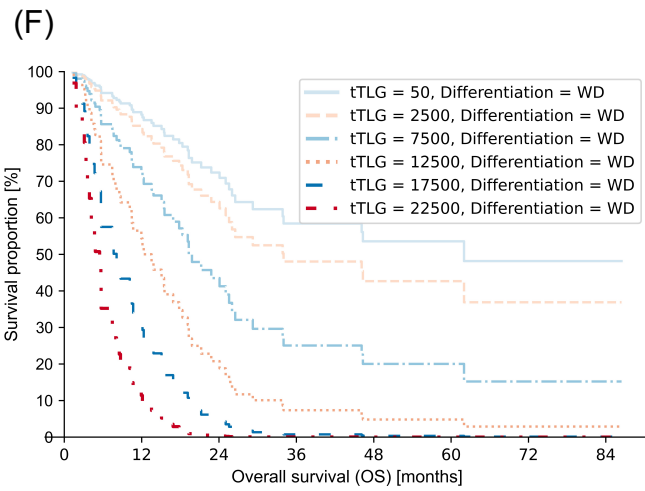

Supplement: Supplementary file 1 — Figure S1 Simulated overall survival (OS) plots from the multivariable Cox regression for the two variables, total metabolic tumour volume (tMTV) and total total lesion glycolysis (tTLG). The simulated survival curves show the effect of varying the variables “tMTV” and “tumour differentiation” whilst setting the variable “SUVmax” to its median value. This to illustrate the impact of a covariate given the Cox regression model. Note that these simulated OS curves include the same number of patients (n = 66) and events (n = 57), whilst in reality the patients and events are distributed between the different curves as one would see in a Kaplan–Meier plot. A, The solid blue line shows the predicted OS when tMTV is equal to the median value (66 cm3) in the low metabolic group and the tumour differentiation is well‐differentiated (WD). Similarly, the dashdotted light blue line shows the OS when tMTV is equal to the median value (564 cm3) in the high metabolic group and the tumour differentiation is WD. The dashed red line and dotted gold line show the OS in both the low and high metabolic groups, respectively given the tumour differentiation is poorly differentiated (PD). B, Shows exactly the same thing as in (A), but for the variable tTLG. C, D; Shows the effect of varying the value of tMTV and tTLG over a whole range of values given that the tumour differentiation is PD. E, F; Similarly, these graphs show exactly the same as in (C) and (D), but given that the tumour differentiation is WD. [file JNE-34-e13170-s004.pdf]
