# Supplementary material for: Volumetric parameters from [ 18F]FDG PET/CT predicts survival in patients with high‐grade gastroenteropancreatic neuroendocrine neoplasms
Source: J Neuroendocrinol. 2022 Jun 21;34(7):e13170. doi: 10.1111/jne.13170 (PMC9539477; doi:10.1111/jne.13170)
Supplement: Supplementary file 2 — Table S1 Scanner parameters – grouped by total MTV and total TLG [file JNE-34-e13170-s001.pdf]

Supplemental Table 1 Scanner parameters – grouped by total MTV and total TLG

	Missing	Overall	Total MTV Group Low	Total MTV Group High	P-value *p*	Total TLG Group Low	Total TLG Group High	P-value *p*
Total, *n*		66	33	33		33	33	
Height [cm], mean (SD)	1	172.5 (9.2)	173.1 (8.1)	172.0 (10.3)	0.630	173.5 (8.2)	171.6 (10.2)	0.423
Weight [kg], mean (SD)	0	76.6 (14.2)	78.3 (12.6)	75.0 (15.6)	0.355	77.5 (11.0)	75.8 (16.9)	0.622
Dose [MBq], mean (SD)	0	256.1 (60.1)	258.5 (51.5)	253.6 (68.3)	0.745	258.7 (48.9)	253.5 (70.2)	0.731
Time from injection to scan [min], mean (SD)	0	65.8 (11.2)	67.2 (14.2)	64.5 (7.2)	0.319	67.8 (14.4)	63.9 (6.4)	0.172
Blood glucose level [mmol/L], mean (SD)	1	6.0 (1.6)	6.0 (1.0)	5.9 (2.0)	0.890	5.9 (1.1)	6.0 (2.0)	0.886
Reconstruction parameters, *n* (%)	0				0.672			0.672
Iterative		6 (9.1)	2 (6.1)	4 (12.1)		2 (6.1)	4 (12.1)	
PSF reconstruction + ToF		60 (90.9)	31 (93.9)	29 (87.9)		31 (93.9)	29 (87.9)	
Matrix size, *n* (%)	0				0.937			0.937
168 x 168		4 (6.1)	2 (6.1)	2 (6.1)		2 (6.1)	2 (6.1)	
200 x 200		9 (13.6)	5 (15.2)	4 (12.1)		5 (15.2)	4 (12.1)	
400 x 400		53 (80.3)	26 (78.8)	27 (81.8)		26 (78.8)	27 (81.8)	
FWHM, *n* (%)	2				1.000			1.000
2.0		60 (93.8)	31 (93.9)	29 (93.5)		31 (93.9)	29 (93.5)	
5.0		4 (6.2)	2 (6.1)	2 (6.5)		2 (6.1)	2 (6.5)	
Number of iterations, *n* (%)	2				1.000			1.000
2.0		60 (93.8)	31 (93.9)	29 (93.5)		31 (93.9)	29 (93.5)	
4.0		4 (6.2)	2 (6.1)	2 (6.5)		2 (6.1)	2 (6.5)	
Number of subsets, *n* (%)	2				1.000			1.000
8.0		4 (6.2)	2 (6.1)	2 (6.5)		2 (6.1)	2 (6.5)	
21.0		60 (93.8)	31 (93.9)	29 (93.5)		31 (93.9)	29 (93.5)	
Scanner type, *n* (%)	0				0.356			0.356
GE Discovery 690		2 (3.0)		2 (6.1)			2 (6.1)	
Siemens Biograph 64		4 (6.1)	2 (6.1)	2 (6.1)		2 (6.1)	2 (6.1)	
Siemens Biograph mCT		60 (90.9)	31 (93.9)	29 (87.9)		31 (93.9)	29 (87.9)	

SD, standard deviation; MTV, metabolic tumour volume; TLG, total lesion glycolysis; PSF, point spread function; ToF, time of flight; FWHM, full width at half maximum; GE, general electric
