# Supplementary material for: Volumetric parameters from [ 18F]FDG PET/CT predicts survival in patients with high‐grade gastroenteropancreatic neuroendocrine neoplasms
Source: J Neuroendocrinol. 2022 Jun 21;34(7):e13170. doi: 10.1111/jne.13170 (PMC9539477; doi:10.1111/jne.13170)
Supplement: Supplementary file 3 — Table S2 Python packages used for data wrangling and data analyses [file JNE-34-e13170-s002.pdf]

Supplemental Table 2 Python packages used for data wrangling and data analyses

Package name	Package version
jupyterlab-server	2.2.0
jupyterlab	3.0.7
pandas	1.2.1
pandas-profiling	2.9.0
numpy	1.19.2
scipy	1.6.0
missingno	0.4.2
scikit-learn	0.23.2
tableone	0.7.10
statsmodels	0.12.1
lifelines	0.25.9
matplotlib	3.3.2
seaborn	0.11.1
