# Supplementary material for: Volumetric parameters from [ 18F]FDG PET/CT predicts survival in patients with high‐grade gastroenteropancreatic neuroendocrine neoplasms
Source: J Neuroendocrinol. 2022 Jun 21;34(7):e13170. doi: 10.1111/jne.13170 (PMC9539477; doi:10.1111/jne.13170)
Supplement: Supplementary file 4 — Table S3 Re‐evaluated histology – grouped by total MTV and total TLG [file JNE-34-e13170-s003.pdf]

Supplemental Table 3 Re-evaluated histology – grouped by total MTV and total TLG

			Total MTV Group			Total TLG Group		
Variable name	Missing	Overall	Low	High	P-value *p*	Low	High	P-value *p*
Total, *n*		66	33	33		33	33	
Re-evaluated, n (%)	0				1.000			0.492
Yes		64 (97.0)	32 (97.0)	32 (97.0)		33 (100.0)	31 (93.9)	
No		2 (3.0)	1 (3.0)	1 (3.0)			2 (6.1)	
Re-evaluation of, n (%)	2				0.203			0.644
Primary tumour		38 (59.4)	22 (68.8)	16 (50.0)		21 (63.6)	17 (54.8)	
Metastasis		26 (40.6)	10 (31.2)	16 (50.0)		12 (36.4)	14 (45.2)	
Biopsy location, n (%)	2				0.558			0.528
Oesophagus		8 (12.5)	6 (18.8)	2 (6.2)		6 (18.2)	2 (6.5)	
Gastric		3 (4.7)	1 (3.1)	2 (6.2)			3 (9.7)	
Pancreas		2 (3.1)	1 (3.1)	1 (3.1)		1 (3.0)	1 (3.2)	
Liver metastasis		20 (31.2)	9 (28.1)	11 (34.4)		10 (30.3)	10 (32.3)	
Small intestine		1 (1.6)		1 (3.1)			1 (3.2)	
Colon		11 (17.2)	7 (21.9)	4 (12.5)		6 (18.2)	5 (16.1)	
Rectum		13 (20.3)	7 (21.9)	6 (18.8)		8 (24.2)	5 (16.1)	
Peritoneum		3 (4.7)	1 (3.1)	2 (6.2)		1 (3.0)	2 (6.5)	
Lymph node		2 (3.1)		2 (6.2)		1 (3.0)	1 (3.2)	
Unknown		1 (1.6)		1 (3.1)			1 (3.2)	
Chromogranin A staining, median [Q1,Q3]	5	90.0 [0.0,100.0]	95.0 [11.2,100.0]	85.0 [0.0,100.0]	0.246	95.0 [22.5,100.0]	82.5 [0.0,100.0]	0.217
Synaptophysin staining, n (%)	4				0.566			0.530
3+		56 (90.3)	28 (93.3)	28 (87.5)		29 (93.5)	27 (87.1)	
2+		5 (8.1)	2 (6.7)	3 (9.4)		2 (6.5)	3 (9.7)	
0		1 (1.6)		1 (3.1)			1 (3.2)	
Architecture, n (%)	3				0.688			0.742
Organoid		11 (17.5)	6 (18.8)	5 (16.1)		7 (21.2)	4 (13.3)	
Trabecular		3 (4.8)	2 (6.2)	1 (3.2)		2 (6.1)	1 (3.3)	
Solid		11 (17.5)	4 (12.5)	7 (22.6)		4 (12.1)	7 (23.3)	
Expancile nests		30 (47.6)	17 (53.1)	13 (41.9)		16 (48.5)	14 (46.7)	
Infiltrative		8 (12.7)	3 (9.4)	5 (16.1)		4 (12.1)	4 (13.3)	
Vessel pattern, n (%)	3				0.907			0.876
Close		34 (54.0)	17 (53.1)	17 (54.8)		18 (54.5)	16 (53.3)	
Distant		29 (46.0)	15 (46.9)	14 (45.2)		15 (45.5)	14 (46.7)	
Co-existing neoplasm or NET, n (%)	2				0.282			0.273
Adenoma		5 (7.8)	1 (3.1)	4 (12.5)		1 (3.0)	4 (12.9)	
Dysplasia		1 (1.6)	1 (3.1)			1 (3.0)		
Adenocarcinoma		1 (1.6)	1 (3.1)			1 (3.0)		
NET								
No		57 (89.1)	29 (90.6)	28 (87.5)		30 (90.9)	27 (87.1)	
Stroma, n (%)	3				0.360			0.212
Yes		33 (52.4)	19 (59.4)	14 (45.2)		17 (51.5)	16 (53.3)	
No		21 (33.3)	8 (25.0)	13 (41.9)		9 (27.3)	12 (40.0)	
Hyalinized		9 (14.3)	5 (15.6)	4 (12.9)		7 (21.2)	2 (6.7)	
Geographic necrosis, n (%)	3				0.901			0.167
Yes		31 (49.2)	15 (46.9)	16 (51.6)		13 (39.4)	18 (60.0)	
No		32 (50.8)	17 (53.1)	15 (48.4)		20 (60.6)	12 (40.0)	

MTV, metabolic tumour volume; TLG, total lesion glycolysis; WD, well-differentiated; PD, poorly differentiated; NET neuroendocrine tumour; MiNEN, mixed neuroendocrine non-neuroendocrine neoplasm; LC, large cell; SC, small cell
